# Modern models of trophic meta-communities

**DOI:** 10.1098/rstb.2019.0455

**Published:** 2020-11-02

**Authors:** Thilo Gross, Korinna T. Allhoff, Bernd Blasius, Ulrich Brose, Barbara Drossel, Ashkaan K. Fahimipour, Christian Guill, Justin D. Yeakel, Fanqi Zeng

**Affiliations:** 1 University of California Davis, Department of Computer Science, 1 Shields Avenue, Davis, CA 95616, USA; 2 Alfred Wegener Institut. Helmholtz Zentrum für Polar und Meeresforschung, Am Handelshafen 12, 27570 Bremerhaven, Germany; 3 Univeristät Oldenburg, Institut für Chemie und Biologie des Meeres, Carl-von-Ossietzky-Strasse 9-11, 26111 Oldenburg, Germany; 4 Helmholtz Institute for Functional Marine Bidiversity, Ammerländer Heerstrasse 231, Oldenburg, Germany; 5 Universität Tübingen, Department of Biology, Auf der Morgenstelle 5, 72076 Tübingen, Germany; 6 German Centre for Integrative Biodiversity Research, Deutscher Platz 5e, 04103 Leipzig, Germany; 7 Institute for Biodiversity, Friedrich Schiller University Jena, Dornburger-Strasse 159, 07743 Jena, Germany; 8 TU Darmstadt, Institut für Festkörperphysik, Hochschulstrasse 6, 64289 Darmstadt, Germany; 9 National Oceanic and Atmospheric Administration, Southwest Fisheries Science Center, 110 McAllister Way, Santa Cruz, CA 95060, USA; 10 Universität Potsdam, Institut für Biochemie und Biologie, Karl-Liebknecht-Strasse 24-25, 14476 Potsdam, Germany; 11 University of California, Merced, School of Natural Sciences, 5200 North Lake Road, Merced, CA 95343, USA; 12 University of Bristol, Department of Engineering Mathematics, Merchant Venturers Building, Bristol BS8 1UB, UK

**Keywords:** dispersal, meta-community, foodweb

## Abstract

Dispersal and foodweb dynamics have long been studied in separate models. However, over the past decades, it has become abundantly clear that there are intricate interactions between local dynamics and spatial patterns. Trophic meta-communities, i.e. meta-foodwebs, are very complex systems that exhibit complex and often counterintuitive dynamics. Over the past decade, a broad range of modelling approaches have been used to study these systems. In this paper, we review these approaches and the insights that they have revealed. We focus particularly on recent papers that study trophic interactions in spatially extensive settings and highlight the common themes that emerged in different models. There is overwhelming evidence that dispersal (and particularly intermediate levels of dispersal) benefits the maintenance of biodiversity in several different ways. Moreover, some insights have been gained into the effect of different habitat topologies, but these results also show that the exact relationships are much more complex than previously thought, highlighting the need for further research in this area.

This article is part of the theme issue ‘Integrative research perspectives on marine conservation’.

## 1. Introduction

Understanding the role of space in the dynamics of ecological communities is a difficult challenge. Different species traverse space at significantly different paces [1], in different dimensions [2] and across vastly different scales [3–5]. Even within a species, different types of movement exist that serve unique purposes [6]: local ranging in search of food, annual migrations or rare long-distance dispersal events in search of a home range, each of which follows their own complex behavioural rules.

Because of the considerable difficulties, many classical ecological models do not model space explicitly. By contrast, spatially explicit models have mostly focused on one population or a narrow set of similar populations. However, there is now growing evidence that community-level processes and spatial constraints influence macroecological patterns [7–10] and that spatial structure shapes community dynamics even when all patches of habitat are identical [11–15].

Today, the need to understand the effect of space on complex ecological systems is more pressing than ever [16,17], as human activity continues to alter the spatial context of ecological interactions through habitat fragmentation and destruction [18,19]. An increased understanding of the effects of space and spatial connectedness on the dynamics of diverse communities not only would allow us to understand anthropogenic impacts better but could also inform mitigation efforts. Particularly, it may be useful in reserve siting [20,21], to identify the most vital areas to protect and inform decisions on how to best connect surviving patches of habitat. In marine systems, it may lead to more holistic resource management and conservation strategies that mitigate effects of overexploitation [22–24].

While ecologists have considered meta-communities since the mid-twentieth century [25,26], groundbreaking work in the 1990s and 2000s sparked many new investigations [21,27–38]. At the same time, advances in network science have led to considerable conceptual progress that has made it easier to formulate and analyse models of meta-communities. Although there is no single modelling framework that comes close to describing a meta-community in all its complexity, over the past decade significant progress has been made with a wide variety of models taking different approaches. Despite the great diversity of ideas, there is now a strong confluence of findings where repeated insights emerge robustly. These findings have also led to new questions, highlighting a strong need for more field observations and laboratory experiments.

**Figure 1.**
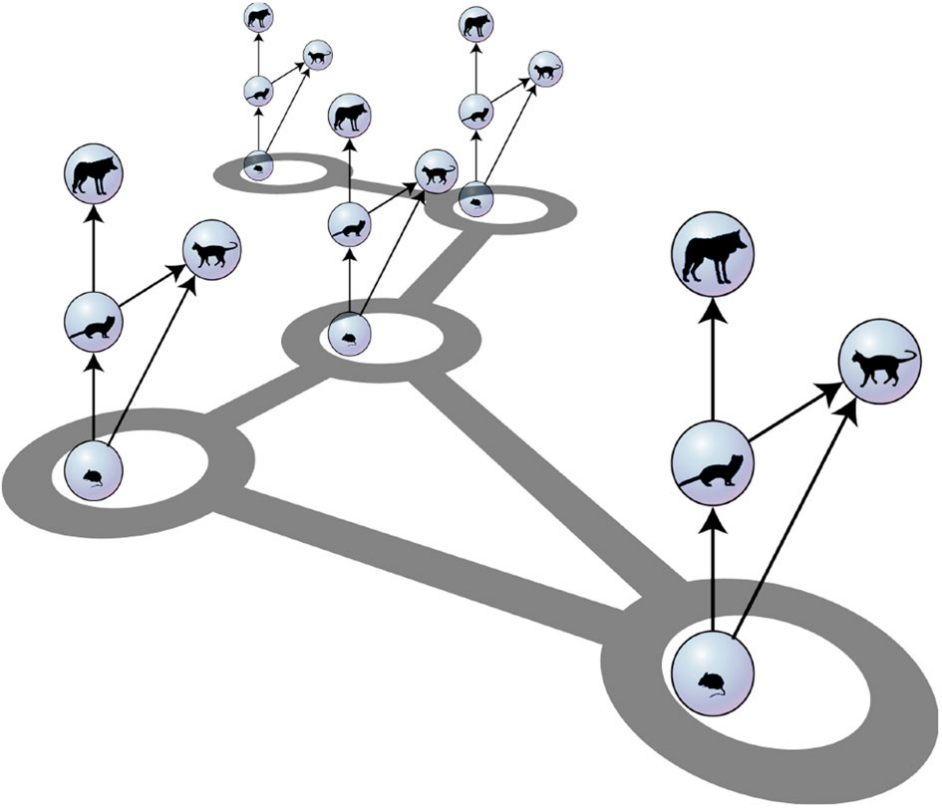
Illustration of a meta-foodweb from Brechtel *et al.* [15]. Distinct habitat patches harbour foodwebs, which interact by dispersal of individuals. (Online version in colour.)

In this review, we focus particularly on meta-foodwebs, i.e. models that describe large communities of trophically interacting populations in a spatially extensive setting (figure 1). We provide an overview of the modelling approaches that have been proposed, before reviewing some key insights and open problems.

## 2. Modelling considerations

The predominant modelling approach for (non-spatial) foodwebs are ordinary differential equation systems (ODEs). In these models, the state of a community is captured by a set of variables such that each variable describes the abundance or biomass density of a population. Assuming that the abundance is sufficiently high to treat it as a continuous variable, the change in time of this variable is then written in the form of an ODE, which accounts for effects of inter- and intraspecific interactions and constraints introduced by the abiotic environment.

Once an ODE system for a community has been formulated, it can then be simulated (i.e. integrated numerically) to produce time series. Alternatively, the model can be analysed with the mathematical tools of dynamical systems theory [39]. A central object in this analysis is the Jacobian matrix. This matrix captures the system’s response to perturbations in the vicinity of stationary states. It thus contains information about the system’s dynamical stability, the dynamics that occur after stability is lost and the impact of parameter changes (press perturbations) [40,41].

In the following, we provide an overview of different approaches to extend foodweb modelling to spatial meta-foodwebs. A summary of these approaches is also shown in table 1.

**Table 1.** Advantages and disadvantages of various modelling approaches. Ordinary differential equation, ODE; partial differential equation, PDE; generalized model with master stability function, GM + MSF; colonization extinction models, C-E; individual-based model, IBM.

model type	advantage(s)	disadvantage(s)	examples
PDE	good representation of continuous space	no long-range dispersal	[42–44]
	analytical approach to spatial pattern formation	simulations are comparatively slow and difficult	
multipatch map	may be more accessible to non-specialists	discrete time models are often less intuitive and are prone to discretization artefacts	[26,45]
	may be advantageous if periodic forcing is important (e.g. year cycle)		
multipatch ODE	powerful framework for fragmented landscapes	larger systems may require numerics	[31,46–49]
	analytical work on stability and responses to perturbations feasible in small systems	modelling heterogeneous systems may require large number of parameters	
random matrix	superior analytical tractability and numerical efficiency	low interpretability of results	[50,51]
		only captures dynamics close to the steady states	
GM + MSF	combines efficiency, tractability and interpretability	only applicable to homogeneous steady states	[15,52,53]
	can reveal which aspects of patch topology impact stability		
C-E	allow deep insights into effects of topology	high degree of abstraction makes is hard to model a specific system	[14,54,55]
	can be studied in highly efficient (event-driven) simulations and a large variety of mathematical approaches		
IBM	highest degree of realism	mathematically intractable	[56–58]
	complex dispersal behaviour is easy to incorporate	difficult to scale to large trophic webs	

### (a) Reaction–diffusion (partial differential equation) models

For a long time, it was felt that the simplest way to add physical space to foodweb models was to consider uniform continuous space (i.e. a vast featureless plain). Instead of one system of ODEs, we now have a system of ODEs in every point in space. This means that the variables are no longer numbers; they become functions of space.

The systems at different points in space are then coupled by some dispersal rule. In the simplest case, this coupling is random diffusion in space, which makes the meta-foodweb model a reaction–diffusion system. In such diffusion dynamics, the immigration rate observed in a point in space is proportional to the second derivative of the density of the respective population, such that the model is mathematically a partial differential equation system (PDE).

PDE simulation is numerically intensive and much care must be taken to avoid numerical artefacts, particularly in strongly nonlinear systems with multiple timescales. Furthermore, including non-local interactions in PDEs is hard, which makes long-range dispersal events hard to model in this framework. An advantage of PDEs is that they provide a very good representation of continuous space and are thus very well suited to modelling the marine environment. Furthermore, it is straightforward to add advective flows to PDEs such that, for instance, ocean currents can be represented easily.

Perhaps the most important advantage of PDE models is that they are a well-established framework for studying the formation of self-organized spatial patterns, such as stationary spot and stripe patterns and travelling waves [42–44]. From an ecological perspective, these pattern-forming instabilities are interesting, as they may explain some of the heterogeneity observed in the environment, and particularly in marine systems [59]. Such heterogeneity can increase the resilience of the system [60,61] by enabling recovery via rescue effects.

In reaction–diffusion PDEs, the threshold parameter values where self-organized pattern formation starts can be computed using a method proposed by Turing [62]. This approach requires additional assumptions, such as identical values of environmental parameter across the space, but can yield deep insights into the conditions for and effects of spatial patterns.

### (b) Multi-patch ordinary differential equation systems

Consider a fragmented landscape consisting of small habitat patches, e.g. multiple reefs or small islands. In such a system, it is intuitive to assume that dispersal within a patch is much faster and more frequent than dispersal between patches. Under these circumstances, we can use variables that denote the population size of a given species in a given patch. Hence, modelling a system with *N* species and *P* patches leads to *N* × *P* variables.

The dynamics of such a patch-based system can be conveniently modelled by a system of ODEs (or even discrete-time maps [26,45]). Like PDE models these ODEs will generally resemble the corresponding non-spatial ODE with the addition of an additional coupling term that connects variables in different patches [31,46–49,63–65]. The multi-patch framework allows the researcher to connect patches in the form of a complex network. The resulting system is a so-called mutlilayer network [66,67] (figure 1). Multilayer networks are currently receiving considerable attention in network science and therefore new analysis tools for this class of system may become available in the near future [67].

The specific pattern of nodes and links in a network is called the network topology. Most papers consider only a small set of topologies for the spatial network [13,68]. Perhaps the simplest option is the fully connected network, where direct dispersal from every patch to every other patch is possible. Another simple choice is to use a completely random network with a given number of links, the so-called Erdős–Rényi random graph. Both the fully connected network and the random graph are small worlds: in these networks any patch can be reached from any other patch in a few steps. Small worlds form whenever long-distance links exist in a network. They have far-reaching implications (see below), which may be undesirable in models.

In general, short-range dispersal in real space will lead to large-world geometries, where distances between randomly chosen patches are on average significantly longer than in comparable small worlds. The simplest models of large worlds are lattices, for example, patches arranged in a one-dimensional line or in a two-dimensional grid. Better models (which avoid some artefacts from lattice geometry) are spatial random graphs, such as the random geometric graph. In this model, patches are assigned random coordinates (say, on a two-dimensional plane). We then connect any pair of patches that are less than a given threshold distance apart by a dispersal route. This leads to a network with a realistic degree of randomness, while retaining a large world character.

In studies that consider only a small number of geographical networks it would be ideal if topologies of real-world systems could be used, but dispersal data are still currently only available for a few species in a few systems. Determining the complete set of dispersal routes in a meta-community between patches is a challenging task. However, in some cases likely routes can be inferred from topography and landscape features [69,70]. Here, marine systems may offer interesting opportunities as for instance larval dispersal is relatively well understood [71] and can (in some cases) be inferred from ocean currents [72]. The results of network inference are typically weighted networks, which include some rare long-distance dispersal events as well as much more frequent short-distance dispersal. Moreover, we can expect the topology of the network to be species-dependent. Owing to the different body sizes and modes of locomotion, some species may be capable of traversing links that are insurmountable to others.

When the local dynamics of the foodweb and the spatial topology have been determined, we still need to decide on a functional form of the coupling terms. In practice, a broad variety of different functional forms is used (table 2). Moreover, we can distinguish two broad classes of couplings. First, in patch-wise dispersal, individuals in a patch make the decision to disperse and then randomly choose one of the available dispersal routes. Second, in link-wise dispersal, we assume that individuals randomly encounter opportunities for dispersal and then use them with a given probability. In the first case, patches of high degree, i.e. those with many dispersal routes, will have a proportionately higher immigration rate, but the same emigration rate as a low-degree patch with the same population size. As a result, patches of high degree will tend to be more crowded. In the second case, patches of high degree have a higher immigration rate, but this is balanced by a correspondingly higher emigration rate.

**Table 2.** Common dispersal strategies in meta-community models. The ‘form’ column describes emigration rates of populations of species *N*_*i*_ from patch *i* to patch *j*. The constants *δ*, *α* and *β* represent different model parameters (see citations); *H*_*i*_ is some measure of habitat quality in patch *i* (e.g. primary productivity); *F*_*i*_ is the *per capita* fitness of species *N*_*i*_; and *P*_*i*_ is the density of a predator in patch *i*.

dispersal strategy	form	examples
diffusion	*δN*_*i*_	[13,15]
habitat-dependent	$\delta N_{i}e^{-\alpha H_{i}}$	[73,74]
fitness-dependent	$\delta N_{i}e^{\alpha (F_{i}-F_{j})}$	[75]
	$\delta N_{i}{\text{e}}^{\alpha (F_{i}-F_{j})}/(1+{\text{e}}^{\alpha (F_{i}-F_{j})})$	[73,74]
density-dependent	$\delta N_{i}^{1+\alpha }$	[76,77]
	$\delta N_{i}{\text{e}}^{\alpha N_{i}}/(1+{\text{e}}^{\alpha N_{i}})$	[73,74]
predator-avoidance	*δN*_*i*_(*αβP*_*i*_/(1 + *N*_*i*_) + (1 − *α*))	[15,78]

Whether patch-wise or link-wise dispersal is the better model depends on the species under consideration. From a mathematical perspective, link-wise dispersal is particularly attractive. Models with identical patches and link-wise dispersal permit homogeneous solutions where we find the identical community with the same population densities in every patch. In analogy to the PDE systems, we can then ask when instabilities exist that lead to spontaneous pattern formation. Following [52,79], we can extend the theory of pattern-forming instabilities in PDEs to multi-patch ODE systems. As Brechtel *et al.* [15] point out, the result is mathematically equivalent to master stability function theory in coupled oscillator systems [80]. In effect, this approach allows the researcher to consider a given foodweb and coupling functions and compute a mathematical criterion that governs in which spatial topologies this foodweb exhibits spontaneous pattern formation. This is very attractive because it is one of the few approaches that enable us to determine which properties of the spatial topology matter, without basing our reasoning on a limited set of examples.

Another feature that makes multi-patch ODE systems attractive is that ODE systems can be simulated relatively efficiently. Although much care has to be taken with the simulation of nonlinear multiple-timescale systems to ensure valid solutions, the simulation of multi-patch ODEs provides a versatile and robust approach to exploring the dynamics of trophic meta-communities. In particular, this can be used to study the effect of patch heterogeneity, where some patches have different size or environmental conditions. For instance, it is known that such spatial heterogeneity can lead to mass effects that alter the outcomes of competition or predation on landscapes [73,81].

### (c) Spectral and generalized models

Despite their complexity, the multi-patch models from the previous section are still ODE systems. Hence, they can in principle be studied with the standard tools of nonlinear dynamics that are widely used to analyse smaller models: write the differential equations, compute the steady states, then compute the Jacobian matrix and its eigenvalues to determine the stability of the steady states. However, if we try to apply this approach to very large systems then difficulties are typically encountered in the second step. Using symbolic mathematics, the steady states can only be computed for small systems with up to approximately four variables. In meta-foodweb models with hundreds or thousands of variables even numerical algorithms frequently fail.

Instead of modelling the differential equations and then computing the Jacobian matrix from their steady states, we can directly formulate a model of the Jacobian matrix. Following in the steps of May’s 1972 paper [82], we can construct an ensemble of random matrices, designed to represent the Jacobians of plausible models. Gravel *et al.* [50] and Moughi [51] extend the random matrix idea to meta-communities. Each patch is modelled as a randomly generated block along the diagonal of the matrix. These blocks are then linked in a random geometry by sparsely placed couplings, representing dispersal. Ecological insights are then gained by studying the eigenvalues of the matrices in the ensemble.

The key advantage of random matrix models is that they can be studied by methods of random matrix theory and hence very general results for the limit of infinite system size can be obtained with pen and paper. The main drawback of random matrix models in general stems from some assumptions that need to be made to make the mathematical tools applicable. A key assumption is that the diagonal entries of the Jacobian can be set to −1. May [82] motivates this by species being self-regulated and argues that any entry could be normalized to −1 using timescale normalization. However, in ODE models, positive diagonal entries are frequently encountered in intermediate predators. Such positive entries cannot be normalized to −1 without altering the system’s dynamics [39].

If positive diagonal elements are present then the framework of random matrix theory can still be applied, but it becomes difficult to extract ecological insight from the results. This is ultimately due to the difficulties in interpreting a given realization of the random matrix as a specific foodweb.

A middle way between random matrix and ODE-based models is provided by so-called generalized models [83]. The central idea of these models is that we can formally write the Jacobian matrices for a broad class of foodwebs. This leads to Jacobian matrices where the remaining unknown parameters have clear interpretations. Moreover, generalized models can be set up such that they incorporate several properties of real-world systems, including plausible foodweb structure, realistic prey-switching, allometric scaling of timescales, plausible nonlinearities of functional responses and biomass turnover rates. In the study of meta-foodwebs generalized models have been used to gain broad insights into the relationships between spatial network topology, in particular dispersal strategies, and the susceptibility of systems to pattern formation [15,53,76,84].

### (d) Colonization–extinction models

A radically different approach to meta-foodwebs was proposed by Pillai *et al*. [14,85]. Following the spirit of Levins’ model [86] and island biogeography [87], colonization–extinction models do not track species’ abundances. Instead, the model only accounts for the presence or absence of a species in a patch. In time this patch occupation changes as local populations go locally extinct or colonize neighbouring patches of the spatial network.

The colonization–extinction models are attractive because they describe some effects that are not captured by differential equation-based models, such as stochastic extinction and the persistence of different communities in different patches [88]. Moreover, the simpler framework of colonization–extinction models enables deep analysis. The models can be studied in extremely fast event-driven simulations, allowing the analysis of large systems and long simulation times [14,54,89–92]. Furthermore, [55] pointed out that colonization-extinction models are mathematically equivalent to co-infection models from network science, and hence can be investigated using the powerful analytical tools that have been developed for these models. Such approaches were leveraged by [54,55], which explored the effect of different topologies, and [93], where a formula for extinction thresholds was derived. However, Barter & Gross [54] showed that some approximation methods provide only rough estimates in spatial networks, owing to their large-world properties. Spatial separation leads to strongly correlated local clusters, which violates widely made assumptions [94].

### (e) Individual-based models

Perhaps the most direct approach to modelling interacting populations in space is individual-based modelling [56–58]. In these models individuals are presented as distinct agents in the model that follow a set of algorithmic rules. Like no other modelling approach, individual-based modelling allows us to directly incorporate observed real-world behavioural patterns into the model.

The drawback of individual-based models is that they are hard to study by methods other than direct simulation, although some promising solutions to this problem are emerging [95]. In individual-based simulations, the simulation code has to keep track of all agents and their internal states. This imposes strong limits on the size of trophic communities that can be studied, because both the timescale of turnover and the number of individuals scale allometrically with trophic level. Studying complex trophic communities thus requires simulating very many small individuals for a long time. To avoid these constraints, it is therefore useful to use individual-based simulations in conjunction with other approaches that allow for the scaling-up of individual-based insights [58]. For example [64] uses patch-based ODE simulations in conjunction with an agent-based model for dispersal events.

### (f) Evolutionary models

Several recent works provided evidence for evolution taking place on the same timescale as population dynamical processes [96–102]. There is thus potential for a complex interplay between evolution, dispersal and local population dynamics [103,104]. For example, it has been predicted that increased dispersal inhibits local adaptation [105]. By contrast, adaptation following colonization can generate eco-evo feedbacks promoting priority effects and monopolization [106,107]. Hence two contrary scenarios are conceivable, adaptation suppressing dispersal or dispersal suppressing adaptation, both of which can occur depending on the relative timescales [108,109].

Adding evolutionary dynamics to trophic meta-communities further increases the complexity in an already complex class of models. To maintain feasibility of these models, we have to make simplifying assumptions on either the foodweb dynamics or the evolutionary process under consideration. This can be achieved by building upon the inherently fast and lean modelling framework of colonization–extinction models [110], limiting the spatial scale or species number [111], limiting changes in species composition to invasion from a fixed species pool in the spirit of island biogeography [9,17,112], or focusing on a very specific system [102] where the parameter range is constrained by observations.

Perhaps the most widely adopted approach for studying the evolution of large trophic communities is that of adaptive dynamics [113,114]. The key idea is to identify one or more traits that undergo continuous change under the selective pressures. Examples for such evolving traits are average adult body masses [115–117], plant defences against herbivores [118], preferred environmental conditions [108] or dispersal abilities [70,119,120]. Adaptive dynamics is then implemented by a series of ‘mutation’ events, by which a new population is introduced to the system as a modification of an existing one. The new population has somewhat different trait values compared with its parent. Whether or not it is able to survive, and if so, whether it is able to replace its parent or whether both coexist (evolutionary branching) depend on the biotic interactions within the foodweb.

The resulting evolutionary meta-communities are typically too complex to be analytically solvable, especially if multiple traits are allowed to coevolve. They are hence often studied using numerically intensive simulation. Considering trait evolution in addition to spatial dynamics increases the required simulation time vastly. Many models are, therefore, still limited in terms of spatial scales and/or consider only few species.

A key trait that deserves special attention is dispersal itself, as it shapes the way a spatial topology is perceived by the species inhabiting it. Predictions concerning foodweb responses to changing spatial environments derived from eco-evolutionary models might therefore contradict earlier results from purely ecological models. Many studies focus on the evolution of dispersal strategies and dispersal syndromes within single species in isolation, as for instance reviewed in [121], whereas theoretical studies on dispersal evolution in meta-communities are still rare.

## 3. Current insights

Although the modelling of large trophic meta-communities is still challenging, the different approaches complement each other nicely, revealing a breadth of perspective. Some of the insights that have been gained draw upon the unique strength of a specific modelling approach while many others emerge in several different types of models.

### (a) Dispersal stabilizes in multiple ways

Almost all models analysed so far identify dispersal between patches as a stabilizing force, in accordance with ecological expectation [25]. Notably, this finding emerges across a wide variety of models including colonization–extinction models [14], random matrix models [50], generalized models [84], patch simulation [47,49,122] and even PDE models [44], although Gramlich *et al.* [84] note that this stabilization is not universal and is rarer in small systems than in large ones.

It is furthermore interesting to note that dispersal stabilizes in different ways, relating to different notions of stability. Perhaps the most intuitive idea is that patch heterogeneity can aid species persistence against large environmental fluctuations, as a species can persist in a favourable patch and later recolonize the less favourable patches. This ‘rescue effect’ has been studied since the 1970s [123]. More recent work suggests that if such rescue effects enable low-population top predators to persist in a given spatial environment, then this might stabilize selective top-down pressures within the foodwebs and hence increase diversity at every trophic level [117].

By contrast, many recent models focus on the simpler setting of identical patches [64]. This choice is partly made to simplify models, but more importantly facilitates the exploration of the many phenomena that already appear in identical-patch networks. In systems of identical patches, it has been shown that dispersal increases the (local asymptotic) stability of steady states and also the probability that a randomly generated network is stable [84], enables the persistence of more diverse communities [14], and increases the resilience of communities (i.e. their ability to recover from perturbations) [50]. In summary, these results highlight a powerful message for conservation: dispersal in general, but particularly the dispersal of top predators, provides a strong stabilizing force. If this ability is lost then deleterious dynamical instabilities become more likely.

### (b) Intermediate level of dispersal is optimal for stability

Several authors have pointed out that the beneficial effects of dispersal on diversity are maximal at intermediate dispersal rates. Intermediate dispersal facilitates both horizontal and vertical diversity, i.e. the diversity within a trophic level as well as the length of food chains, e.g. [14,47,63]. Gravel *et al.* [50] and Gramlich *et al.* [84] note that intermediate values of dispersal yield optimal dynamical stability, and Plitzko & Drossel [49] show that it maximizes robustness, i.e. the percentage of species that survive after undergoing population dynamics. Jansen [124] shows that at intermediate dispersal coupled predator–prey systems desynchronize, which benefits their resilience.

Although very low dispersal rates can sometimes synchronize the dynamics of patches, it is intuitive that very low dispersal rates generally only have a small impact on the ecological dynamics. By contrast, very high dispersal rates are likely to homogenize the system, causing it to behave as a single patch. Thus it is not surprising that the effect of spatial structure is strongest at intermediate dispersal rates.

While the effect of spatial structure on population dynamics is overwhelmingly positive, the effect on evolution is more subtle. In a system of heterogeneous patches, dispersal from a large population in one patch can undermine the ability of smaller populations to adapt to the respective conditions in their patch, owing to genetic swamping. This can occur in systems where the population size in the patches is initially similar. In this case, a spontaneous breaking of the symmetry of population sizes can occur: a small fluctuation leading to an initial disparity in population size can launch the smaller population into a cycle of decline, where smaller population leads to decreasing adaptation, which further reduces population size [102,125]. This phenomenon has been described as a ‘migrational meltdown’.

Although the migrational meltdown seems detrimental to a species it could promote diversity in a multispecies system. It leads to a population that is very well adapted to a particular patch, improving its local resilience, while its low adaption in other patches opens up niches for competitors.

Despite the overwhelming evidence for the beneficial effects of intermediate levels of dispersal, it is still unclear what this means in practice, e.g. in a conservation context. How should we decide whether dispersal is in an intermediate range in a given real-world system [54]?

### (c) Sparse regions provide refuges for generalists

Meta-community dynamics in spatial networks such as random geometric graphs or real-world patch networks behave differently from the more commonly studied models for network topology (Erdős–Rényi random graph, configuration model, Barabasi–Albert model etc.). Barter & Gross [54] show that the difference is due to the existence of long-range connections. The non-spatial models are small worlds, and hence two randomly picked nodes tend to be only a few dispersal steps apart. This means that a species that is established in at least one patch can rapidly recolonize the whole network if conditions are favourable. It also means that the patches behave very similarly, providing few refuges for weaker competitors.

**Figure 2.**
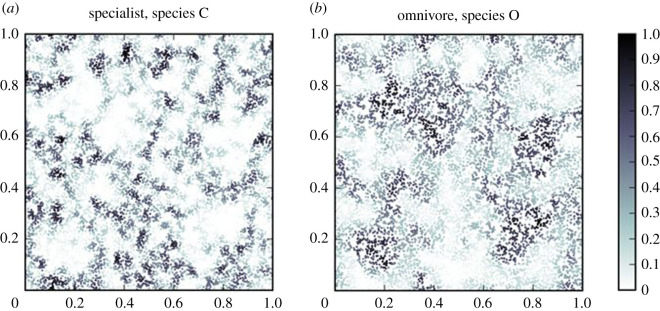
(*a*,*b*) Patch occupation probabilities for a specialist predator and its generalist competitor in a model with 10 000 patches. The generalist occupies regions where the patch density is too low for the specialist to persist. The *x* and *y* axes are normalized spatial coordinates. (from Barter & Gross [54]). (Online version in colour.)

Spatial networks are large worlds. In these networks, it is easy to find pairs of nodes that are separated by significant physical distance and hence a population spreading from one to the other may need many intermediate steps. In random geometric graphs, this leads to the appearance of clusters of nodes (valleys) separated by sparse network regions (ridges). In colonization–extinction models, this leads to higher persistence thresholds, but increases horizontal diversity by providing refuges for generalists [14,54] (figure 2). Similar refuges can be expected in all large-world networks, including even uniform lattices, where lattice edges provide regions of lower effective connectivity [14,89]. In summary, we can say that the large-world nature of spatial networks is important for the persistence of generalists and hence horizontal diversity. From a conservation perspective, this means that we need to be wary of creating long-range connections or artificially dense connectivity (for example by anthropogenic transport along shipping lanes). In addition to the widely recognized risk of bioinvations [126], there is an additional less-recognized risk that increased connectivity turns the system into a small world, triggering a reduction of horizontal diversity.

### (d) Effects of spatial topology are more complex than thought

A central question in many recent studies is which patch structures are most beneficial for the persistence of diverse communities. If a satisfactory answer could be found it would certainly be valuable for conservation as the spatial structure of many systems results from decisions by humans (e.g. which patches of habitat to conserve, where to place wildlife corridors, where to reduce anthropogenic introduction of species etc.).

There is a wide consensus that spatial structure impacts properties such as robustness [49,122,127], stability [15] and diversity [14,50,117]. Moreover, there is widespread evidence that network structure and connectivity impact species on higher trophic levels more strongly [14,55,65,92], which may lead to counterintuitive situations where prey species profit from habitat fragmentation as they experience a release of top-down control [91].

Richhardt *et al.* [122] use principal component analysis of various network metrics to identify metrics that impact the robustness of meta-foodwebs. Their main result is that connectivity increases robustness, which is consistent with widely held beliefs. However, more detailed analysis shows that connectivity, like many network properties, is a double-edged sword. For example, a detrimental effect of high connectivity is that it reduces the network diameter, the typical distance between randomly chosen nodes. This benefits specialists, but may threaten generalists who depend on less-accessible corners of the network to persist [14,54]. Networks with high connectivity thus favour long food chains of specialists, whereas low connectivity networks favour high horizontal diversity of generalists (cf. table 3).

**Table 3.** Qualitative effect of structural network properties.

network property	high	low
connectivity	longer food chains, beneficial to specialists	beneficial for horizontal diversity, beneficial to generalists
degree heterogeneity	high robustness particularly for basal species	higher abundance, benefits apex predators
diameter	good for generalists (horizontal diversity)	longer food chains (vertical diversity)

Another trade-off is observed when it comes to the network’s degree distribution, i.e. how the links are distributed among the nodes. Networks where most links connect to a small number of big hubs are said to be heterogeneous, whereas networks where links are evenly distributed among the nodes are said to be homogeneous. Based on the analogy between meta-population dynamics in colonization–extinction models and the epidemiological susceptible–infected–susceptible (SIS) model, we may suspect that heterogeneous networks are unconditionally advantageous. However, the detailed analysis by Barter & Gross [55] shows that this is not always the case. Very heterogeneous structures promote the persistence of primary producers, but may negatively impact their abundance. By focusing many links on a particular patch we ensure that this patch can be very rapidly recolonized if necessary. Such hub patches thus become reliable bases for the recolonization of the rest of the network. But by concentrating most of the links on few hubs we create a large number of peripheral patches with weak connectivity, which limits the potential to attain high meta-population abundances.

In addition to this heterogeneity trade-off, which can already be observed in a single basal species, Barter & Gross [55] note that heterogeneity affects different species in a food chain differently. The optimal distribution of links thus depends on the trophic level. Generally, slightly more homogeneous networks favour species at higher tropic levels. However, even for top predators the optimal level of heterogeneity is still comparatively high.

### (e) Interplay of speciation, dispersal and extinction produces known spatio-temporal patterns

In contrast to the other models mentioned so far, evolutionary meta-community models include the birth and death of species. Hence they can be used to evaluate macroecological patterns such as species-lifetime distributions, species-abundance distributions or species–area relationships [7]. Neutral meta-community models [128] and competitive meta-community models [9] were shown to give rise to such patterns, but each study focused only on two or three such patterns. Introducing trophic structure gives additional insights, as now species–area distributions and the temporal dynamics of species range depend on trophic level. In a trophic model that does not take into account population sizes [110], it was found that species–area distributions and lifetime distributions both become steeper with increasing trophic level. The most extensive study of this type so far [129] is similar to the Webworld foodweb model [130], and exhibits all of the patterns mentioned above.

## 4. Open questions

In the preceding section, we have listed some areas in which past theoretical work has provided at least tentative answers. In other areas, theoretical progress has led to new questions or highlighted open issues that have much higher system-level relevance than previously thought.

### (a) Quantifying the laws of dispersal

Several papers have pointed out the importance of functional forms of dispersal [73,75]. However, there is still a surprising lack of general laws and principles in this area.

For example, the seemingly small difference between patch-wise and link-wise dispersal (see above) has major consequences for the state of the entire system. Yet, we still know very little about which populations disperse patch-wise or link-wise respectively. Even for single species it is sometimes not clear how individuals make the decision to disperse to a different patch. The same is also true for the rate of dispersal. In the past, authors have used a variety of functional forms for dispersal and different functions have been shown to have significantly different impacts [76,84].

A common assumption is that dispersal decisions can only depend on properties of the source patch as the individual has no information about the destination patch. However, at least for some birds and mammals, it is very plausible that individuals explore potential destination patches as part of their ranging behaviour, before committing to dispersal. Other species may pick up clues about potential destination patches from conspecifics [131]. A very recent paper by Mougi [51] shows that intelligently targeted dispersal may have a significant impact on persistence, but besides this study and a two-patch model by Abrams & Ruokolainen [75] the effect of targeted dispersal in foodwebs remains largely unexplored.

For large meta-foodweb models, we should ideally have allometric scaling relationships that hold across species, but so far even very basic properties of such laws remain unclear [58]. On the one hand, evidence suggests that dispersal rate scales positively with body mass, owing to the superior locomotive capabilities of large-bodied species [1]. But there are also examples of systems where predators have lesser dispersal ability than their prey [132]. More generally, in many species, dispersal occurs owing to juveniles dispersing to establish a home range which they then occupy for the rest of their life [133], leading to negative scaling relationships with lifespan and hence body size.

A promising route to understanding dispersal across species is to explore the evolutionary mechanisms that have shaped it, but general laws have not yet emerged. For example, using an individual-based modelling approach, Chaianunporn & Hovestadt found that parasitism promotes dispersal of hosts and parasites, while mutualism tends to reduce dispersal in both partners [134]. Along similar lines, but using an adaptive dynamics approach, Pillai *et al.* [120] showed that the evolutionary response of dispersal rates to patch extinctions differed between predators and their prey, and Wickman *et al.* [135] derived equations for the strength of selection in meta-communities.

Because of the importance of dispersal rates for meta-community dynamics, we feel there is a great need for future experiments and field observations in this area. In the past, progress was hampered by the difficulty associated with measuring the dispersal across species. But recent studies such as the work by Sivakoff *et al.* [136] suggest that molecular methods may alleviate some of this difficulty in field studies.

In marine systems certain forms of dispersal, such as passive larval dispersal, offer a particularly good opportunity to understand the dispersal network of at least a fraction of a foodweb with very good accuracy [71,72].

### (b) Modelling multilayer networks: what is a patch?

A present bottleneck in the study of meta-foodwebs is that it is still difficult to relate theoretical results to real-world systems. This difficulty arises partly owing to simplifications that are commonly made in models to make progress in the face of considerable complexity.

One such assumption that is widely made is that different species perceive essentially the same network of patches (although they may traverse it at different rates). In many real-world systems, this assumption is violated, a single bush in a forest may be perceived as a patch by an insect living in it, but is just a feature within a patch to a fox walking by, while the whole forest appears as a single patch to an eagle flying overhead. It is tempting to just assign higher dispersal rates to the larger more mobile species. However, this ignores the fundamentally different biology of the different types of movement—the eagle’s circling is part of ranging behaviour in search of prey, not dispersing to new patches where an independent subpopulation of eagles are established. So far, we are only aware of one model [53] that takes this distinction into account.

Unsurprisingly, the models that have revealed the most general insights into the stabilizing effects of dispersal and its dependence on network topology are the strongly stylized colonization–extinction models in the spirit of Pillai *et al.* [14]. The strong simplifications made in these models make them also some of the hardest models to relate back to the real world (although Lafferty & Dunne [88] illustrate how they can be made relevant to concrete systems). Also, some other fairly abstract approaches to the problem have started to emerge [137,138]. At this stage, a crucial step forward could be made by a large project to quantify the foodweb and dispersal of all relevant populations in a specific example system. While the effort for such a project would be considerable, it could provide modelling with a much-needed benchmark.

### (c) What are the precise impacts of spatial network structure?

While the studies reviewed above have revealed some useful general insights into the effect of spatial structure, the results have also made clear that our current understanding barely scratches the surface.

Many phenomena observed in meta-foodwebs are still very counterintuitive. Koelle & Vandermeer [48] show, for example, that increasing the dispersal between two patches can induce asynchrony in their dynamics. This particular result can be understood using the master stability function approach developed in [15]. Moreover, this approach can even yield a general criterion regarding all possible spatial structures in which a given foodweb will behave asynchronously. However, for all but the simplest foodwebs these conditions rapidly become so complex that they have so far defied easy classification or intuitive understanding.

The same is even true for colonization–extinction and random matrix models. While conceptually simple and efficient to analyse, the complexity of the results is such that it is hard to systematize; while it is easy to understand the dynamics of a given model, the progress in extracting general laws, rules or intuitions that hold across different systems is slow [54].

In this area, there is reasonable hope that future advances in theory will lead to significant ecological insights in the future. Particularly, the study of multilayer networks [66,67] and epidemic processes on networks [139] could establish much needed tools for the modelling of meta-foodwebs.

## 5. Conclusion

In this review, we have discussed some recent models of meta-foodwebs, trophic meta-communities that combine spatial dispersal with complex trophic interactions. Understanding the dynamics of these systems is interesting, because it has a strong effect on macroecological patterns, and pressing, because it concerns the maintenance of diverse communities in the face of habitat loss, fragmentation and global change.

Owing to the complexity of meta-foodwebs, strong simplifications are necessary to make theoretical progress, and these simplifications in turn make it difficult to relate insights back to the real world. However, meta-foodwebs are now studied with a broad variety of different modelling frameworks. This has led to a confluence of insights where models reveal complementary perspectives and a set of general principles has just started to emerge.

The results that have been obtained have confirmed some basic intuitions but they have also revealed a wide range of counter-examples and counterintuitive effects. While significant progress has been made, this progress seems to have revealed merely the tip of the iceberg. For example, connectivity and network heterogeneity generally promote diversity but they are not beneficial in all cases or to all species in a system.

Seen collectively, the past results make it clear why models of trophic meta-communities are necessary. By studying non-spatial models of foodwebs, we neglect the important impacts of spatial structure on population dynamics, and forgo the opportunity to understand the effect of population dynamics on macroecological patterns. Conversely, by considering only single meta-populations we run a risk of confusing effects that are beneficial to a single species with effects that are beneficial for diversity. Current results suggest that systemic benefit is often gained at the expense of some species that are strong competitors.

While the complexity of meta-foodweb models poses tough challenges, there is good reason for hope that significant future progress can be made in this area. This progress will most likely emerge from a combination of refinement of theoretical methods, extensive numerical studies, laboratory experiments and large-scale field observations that leverage molecular methods and/or remote sensing technologies.

## Data accessibility

This article has no additional data.
